# Molecular Mechanistic Insights into the Endothelial Receptor Mediated Cytoadherence of *Plasmodium falciparum*-Infected Erythrocytes

**DOI:** 10.1371/journal.pone.0016929

**Published:** 2011-03-17

**Authors:** Ang Li, Tong Seng Lim, Hui Shi, Jing Yin, Swee Jin Tan, Zhengjun Li, Boon Chuan Low, Kevin Shyong Wei Tan, Chwee Teck Lim

**Affiliations:** 1 Singapore-MIT Alliance for Research & Technology (SMART), Singapore, Singapore; 2 Singapore Immunology Network (SIgN), Agency for Science, Technology and Research (A*STAR), Singapore, Singapore; 3 Division of Bioengineering and Department of Mechanical Engineering, National University of Singapore, Singapore, Singapore; 4 Department of Microbiology, Yong Loo Lin School of Medicine, National University of Singapore, Singapore, Singapore; 5 NUS Graduate School for Integrative Sciences and Engineering, National University of Singapore, Singapore, Singapore; 6 Mechanobiology Institute, National University of Singapore, Singapore, Singapore; 7 Department of Biological Sciences, National University of Singapore, Singapore, Singapore; 8 Infectious Disease Program, Life Sciences Institute, National University of Singapore, Singapore, Singapore; Université Pierre et Marie Curie, France

## Abstract

Cytoadherence or sequestration is essential for the pathogenesis of the most virulent human malaria species, *Plasmodium falciparum* (*P. falciparum*). Similar to leukocyte-endothelium interaction in response to inflammation, cytoadherence of *P. falciparum* infected red blood cells (IRBCs) to endothelium occurs under physiological shear stresses in blood vessels and involves an array of molecule complexes which cooperate to form stable binding. Here, we applied single-molecule force spectroscopy technique to quantify the dynamic force spectra and characterize the intrinsic kinetic parameters for specific ligand-receptor interactions involving two endothelial receptor proteins: thrombospondin (TSP) and CD36. It was shown that CD36 mediated interaction was much more stable than that mediated by TSP at single molecule level, although TSP-IRBC interaction appeared stronger than CD36-IRBC interaction in the high pulling rate regime. This suggests that TSP-mediated interaction may initiate cell adhesion by capturing the fast flowing IRBCs whereas CD36 functions as the ‘holder’ for providing stable binding.

## Introduction

It has been observed since a century ago [1] that only early ring stage *Plasmodium falciparum* (*P. f falciparum*) infected red blood cells (IRBCs) appear in peripheral circulation whereas trophozoite and schizont stage IRBCs are sequestered within the capillaries of various organs including heart, gut, liver, lungs, kidney and brain [2], [3]. Such sequestration is mainly caused by the adherence of infected erythrocytes to the endothelial cells lining blood vessels, a process termed ‘cytoadherence’. This phenomenon has been considered central to the pathology of potentially lethal *P. falciparum* infection as it would prevent IRBCs from traveling to the spleen sinusoids where they would be cleared due to their loss of deformability. Also, sequestration in deep microvasculature provides a microaerophilic venous environment which is more suited for parasite development. However, to the human host, cytoadherence and consequent accumulation of masses of late stage infected cells in the capillaries may obstruct microcirculatory blood flow, leading to metabolic dysfunction and locally diminished tissue perfusion and finally organ failure. Clinical studies have shown positive correlations between the level of cytoadherence in the cerebral blood vessels and the severity of the disease [4], [5], [6].

Direct evidence of cytoadherence comes from histological examinations of the microcirculation in blood vessels from cerebral malaria patients in which large amount of late stage parasitized cells accumulate and sequentially perturb or fully obstruct blood flow [7]. A close look at these cytoadherent infected cells using transmission electron microscopy reveals that the ‘knob’ structures are the focal adherent points via which the infected cell contacts with the endothelial cells lining the blood vessel [8], [9]. These surface protrusions have been well characterized morphologically [10], [11] and been suggested to be utilized by the parasite to enhance the binding efficiency in the hydrodynamic flow environments [12]. At the sub-cellular and molecular level, numerous studies have been conducted to examine the molecular basis of cytoadherence with a number of *in vitro* systems. It has been shown that more than 10 receptors expressed on the surface of vascular endothelial cells in various organs and more than five ligands on the infected cell membrane may be involved [13]. Although qualitative studies have identified the diverse array of molecules that could be involved in the adhesive interactions, their relevant roles *in vivo* are difficult to gauge as the kinetics and dynamics of these interactions vary dramatically [14]. For example, CD36, thrombospondin (TSP), chondroitin sulfate A (CSA) and intercellular adhesion molecule-1 (ICAM-1) are widely expressed on the surface of endothelium in various organs and found to be able to mediate stable bindings in static assay. Thus, they are thought to be the major receptors mediating cytoadherence [15], [16], [17], [18], [19]. However, their dynamic binding properties under flow conditions are different: CD36 and CSA form stable bindings whereas ICAM-1 mediates rolling adhesion and TSP alone fails to form stable bonds under flow conditions [14]. This indicates that the kinetic properties of these potential cytoadherent molecules are critical for understanding the molecular mechanisms behind IRBCs-endothelial interactions. Also, to date, most studies have focused on a population of cells and few groups have quantified the accurate binding strength between specific molecules and single infected cells. Thus, quantifying molecular binding strength on a single IRBC is important to provide information on how cell-cell adhesion is controlled at the molecular level and further contribute to our understanding of the pathological roles of the specific molecules which are involved in IRBC-endothelium interactions.

Over the past decade since the advent of atomic force microscopy (AFM), it has become possible to measure single-molecule interaction forces at the pico-newton level [20], [21]. Based on the fact that the application of an external pulling force to an intermolecular bond will reduce the chemical activation energy, and therefore accelerate bond dissociation driven by thermal fluctuation [22], [23], the force and the intrinsic energy profile associated with ligand-receptor interactions can be determined with fairly high resolution.

In this paper, AFM based single-molecule force spectroscopy was applied to study the binding kinetic properties of two host cytoadherent molecules, CD36 and TSP with IRBCs of *P. falciparum* 3D7 cell line at physiological temperature. Force spectra of CD36 against IRBCs and TSP against IRBCs were collected under well controlled conditions. We found significant differences in the intrinsic kinetic properties of these two endothelial receptors from which we could interpret their pathophysiological functions based on their biophysical characteristics.

## Results

### AFM Tip Functionalization

**Figure 1:**
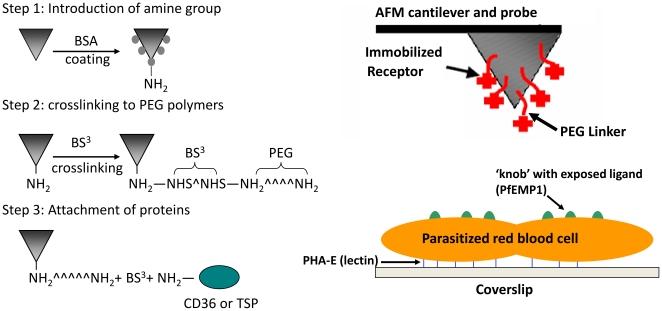
Schematic illustration of AFM tip functionalization chemistry and experimental configurations.

**Figure 2:**
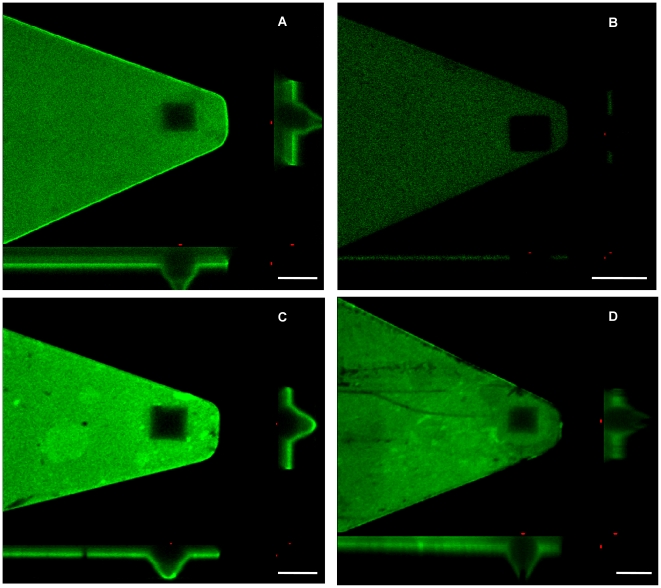
Three dimensional confocal images of functionalized AFM tips stained with antibody conjugated with fluorescent dyes. (A) Freshly prepared AFM tip with TSP conjugated. (B) Unmodified AFM tip as a control. (C) Used but still functional tip. (D) Used and ‘dead’ tip. Images were taken using Nikon N1 confocal microscope equipped with a 100X oil immersion objective. Scale bars represent 5 µm.

Figure 1 shows the schematic illustration of different steps of AFM tip functionalization and experimental configurations as described in the methods section. CD36/TSP molecules were attached to the tips via steps of covalent bonding while the IRBCs were immobilized on glass substrates via strong interaction between glycoprotein and PHA-E lectin. Flexible PEG linkers were for the purpose of reducing nonspecific interactions and increasing single molecule binding probability since they are chemically inert and allow functionalized molecules to freely reorient for recognition binding [24]. Figure 2A shows a freshly prepared TSP-functionalized AFM cantilever labelled with fluorescent antibodies and examined under confocal microscopy. It was clearly shown that the whole cantilever as well as the pyramid shaped tip was fully covered with TSP molecules. On the other hand, in Fig. 2B, an unmodified tip (bare tip without treatments in Fig. 1) that had been incubated with the same fluorescent antibodies showed much weaker fluorescence on the cantilever and a totally dark tip region which indicated no nonspecific adsorption of antibodies on the tip. This confirmed the successful adsorption of receptors onto the AFM tips through specific surface conjugation methods and the density of the surface coated receptors was sufficient for single molecule binding detection. Furthermore, a functionalized tip that had been used for collecting ∼400 force curves showed no decay in the fluorescence intensity (Fig. 2C). By contrast, an extensively used tip that had collected ∼1500 force curves and had been shown to be unable to generate specific binding events exhibited a black opening at the tip’s apex (Fig. 2D).

### Control Experiments

To ensure that the measured forces resulted from specific interactions between tip coated receptors and the cell expressed ligands, it is important to check all the possible combinations of different stages of tip and sample treatments to rule out possible artifacts resulted from nonspecific interactions.

In the control experiments, combinations of fresh tips, semi-functionalized tips (step 1 or step 2 functionalized tips as shown in Figure 1) and fully functionalized tips versus PHA-E coated glass surface, healthy RBCs (hRBCs) and infected RBCs were tested as listed in Table 1. The results confirmed that the tip coated receptor molecules did not interact non-specifically with hRBCs. On the other hand, cell surface ligands on IRBCs did not interact with non-fully functionalized tips (neither BSA nor PEG linkers interact with IRBCs). Thus, only tip coated specific receptors interact with IRBCs surface ligands in our experimental configurations. Additionally, blocking antibodies against CD36 was introduced to block the interaction of CD36 to IRBCs as a further control. It was shown that the binding frequency (defined as the number of force curves showing binding events out of the whole set of force curves) decreased significantly (by 69.1%, *P*<0.001) after the functionalized tips had been incubated with blocking antibodies, indicating the molecular interaction observed was specifically mediated by CD36. Another control experiment involved using recombinant cysteine-rich interdomain region (CIDR) peptide which is one of the best identified CD36 binding ligand on the surface of IRBCs. It was shown that interaction frequency between purified CD36 and recombinant CIDR peptides was also reduced significantly (by 71.4%, *P*<0.001) using the blocking antibody but the interaction between CD36 and IRBCs was only affected by 44.5% (*P* = 0.0835) with high concentration (200 µg/ml or 9 µM) of free CIDR peptides added in the solution. It was also noted that the binding force between CD36 and IRBCs decreased by a level of 30-50% after free CIDR was added. It might indicate a non-CIDR binding site existing on the surface of IRBCs.

**Table 1:** Control experiments and results.

Molecules on tip	Molecules or cells on substrate	Results
Fresh tip	hRBCs/IRBCs	No interaction
BSA* coated tip	hRBCs/IRBCs	No interaction
PEG† linkers	hRBCs/IRBCs	No interaction
TSP/CD36	PHA-E‡	Some interaction but much shorter extension compared with that collected on cells
TSP/CD36	hRBC	No interaction
CD36 with blocking antibody	IRBCs	Frequency of interaction was significantly reduced from 35.5% to 11.0% (*P*<0.001) after blocking
CIDR	CD36 with blocking antibody	Frequency of interaction was significantly reduced from 28.7% to 8.2% (*P*<0.001) after blocking
CD36 with CIDR	IRBCs	Binding frequency dropped from 16.4% to 9.1% (*P* = 0.0835) after addition of free CIDR molecules with final concentration of 200 µg/ml

All the possible combinations of different levels of functionalized AFM tips against different samples were tested to exclude non-specific interaction induced artifacts.

*BSA: Bovine serum albumin

† PHA-E: Phytohemagglutinin E

‡ PEG: Polyethylene glycol

### Kinetic parameters extrapolated from the force spectra

**Figure 3:**
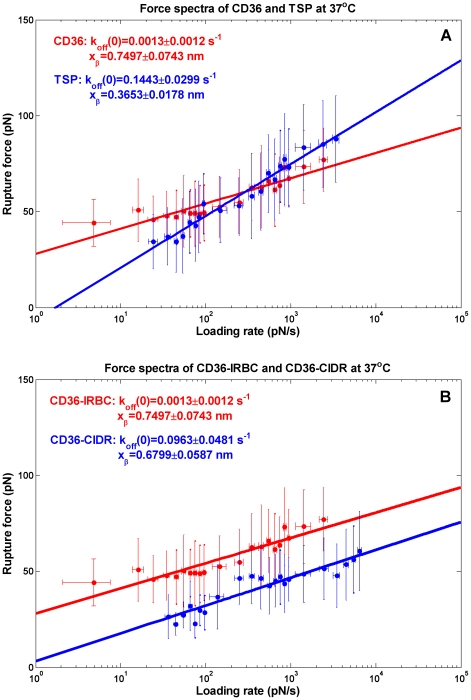
Force spectra and the extrapolated kinetic parameters. (A) CD36-IRBCs interaction and TSP-IRBCs interaction at physiological temperature (B) CD36-IRBCs interaction and CD36-CIDR interaction at physiological temperature. Mean rupture forces within each binned window of loading rates were used as pooled measures to fit to Bell-Evan's model for the characteristic dynamic force spectra reconstruction.



The force spectra obtained in our study were shown to follow the theoretical prediction from Bell-Evans model (Fig. 3A, B) [23], [25]:

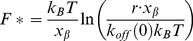
(1)where F* is the most probable rupture force, *r* is the loading rate, *x_β_* is a thermally averaged distance along the direction of the applied force, which denotes the displacement of the unbonding transition state along the reaction coordinate or termed reactive compliance, *k_off_*(0) is the unstressed dissociation rate, *k_B_* is the Boltzmann constant and T is the absolute temperature. Thermodynamically, *k_off_*(0) describes the rate at which spontaneous unbinding driven by thermodynamics occurs while *x_β_* describes the effective molecule length over which the binding molecules disengage from each other upon unbinding. This equation shows that F* increases linearly with the natural logarithm of the mean loading rate *r*. Thus, from the experimental data, we could obtain the intrinsic unstressed dissociation rate and the free reactive compliance by fitting the binned force spectra with a linear line using the least-squares method on the logarithm scale of the loading rate (Fig. 3). The slope of the line was assigned 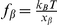 in which *f_β_* is thermally activated rupture force and 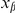 is the free reactive compliance. Moreover, the bond life time *τ*(0) and the dissociation rate 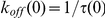
of the bond extrapolated to zero force could also be derived from eq. (1).

### Kinetic comparison between CD36 and TSP

The two molecules were selected based on their distinct binding kinetics under flow conditions [14] as well as their abilities to bind to our platelet selected 3D7 cell line. The single molecule force spectra of CD36 and TSP obtained at physiological temperature is plotted in Fig. 3A. As shown in the figure, the characterized force spectra for CD36 and TSP were distinctly different. The derived *k_off_*(0) from Bell-Evans model for CD36 (mean at 0.0013 s^-1^) was about hundred times smaller than that of TSP (mean at 0.1443 s^-1^) and *x_β_* of CD36 (mean at 0.7493 nm) was about as twice as *x_β_* of TSP (mean at 0.3653 nm) (both *P*<0.001). These data indicate that at physiological temperature, a CD36 associated bond is much more stable but more sensitive and less resistant to external forces than that of a TSP associated bond.

### Force spectra of CIDR compared to IRBCs at 37°C

As the CIDR domain of *Pf*EMP1 is one of the best characterized binding domains to CD36, we examined the results obtained on living cells using a purified system comprising the recombinant CIDR peptide covalently attached to glass surface as described in methods section. Force spectra of CD36-IRBCs interaction and CD36-CIDR interaction are shown in Fig. 3B. The two fitted lines were nearly parallel to each other and the CD36-CIDR interaction force appeared consistently smaller than that of CD36-IRBCs interaction force at given loading rates, which indicates that the CD36-CIDR interaction is not strong enough to solely constitute CD36-IRBCs interaction. Since we calculated loading rate using the effective spring *k_eff_* in our analysis, the difference in the cell stiffness and substrate stiffness should have been corrected or adjusted and not contribute to the different spectra observed here. Although one of the causes may be theconformational change of CIDR from their native state in a purified system, it is also possible that there may be another binding molecule(s) interacting with CD36 together with CIDR on the cell surface, which was supported by the control experiment results as well. The dissociation rate of CD36-CIDR interaction (mean at 0.0963 s^−1^) was about 75 times that of CD36-IRBCs interaction (mean at 0.0013 s^−1^) while the difference of the free reactive compliances of the two (mean at 0.6799 nm and 0.7497 nm, respectively) were less than 10%, though statistically significant (*P* = 0.002).

## Discussion

In this paper, single-molecule force spectroscopy technique has been applied to explore the binding kinetics of CD36 and TSP with IRBCs at physiological temperature. The unbinding forces between AFM tip covalently bound receptors and cell surface ligands on IRBCs have been measured statistically at different pulling speeds. The loading rates covered in our experiments (10–1,000 pN/s) are equivalent to the magnitude of physiological shear stresses that a moving cell is experiencing in the post-capillary bloodstream [26], [27] and the unbinding forces appear consistent with previously reported values estimated from flow experiments [13]. However, no matter how precise the measurement is, unbinding forces are always forming a distribution and are dependant critically on how fast the bonds are loaded as discussed in detail by Merkel et al [28]. In short, the force acting on the bond is generated by the bending of the AFM cantilever, which is not constant but increasing with time during the pulling process. Most importantly, the rupture of the bond is driven by the thermally activated kinetics and facilitated by the external mechanical forces. This gives rise to a reciprocal relation between bond lifetime and measured rupture forces: bonds under slow loading rates have longer lifetime but exhibit smaller strength, whereas bonds under fast loading rates have shorter lifetime but exhibit stronger strength. By measuring the unbinding forces over a range of loading rates, the response of adhesion bonds to external forces under different force loading rates could be mapped and the chemical energy landscape traversed in force-driven dissociation could be revealed [29].

At physiological temperature, it has been clearly shown that the dissociation kinetics of TSP and CD36 are significantly different. TSP can withstand high level of forces at high loading rate regime but the unbinding force decays rapidly when the loading rate is reduced. By contrast, CD36 can maintain a considerable level of binding force even at low loading rate regime (Fig. 3A). Therefore, TSP could behave as a transient connector whereas CD36 could behave as a persistent connector, since their dynamic force spectra appeared very similar to that defined as transient connectors and persistent connectors [30]. This is important for interpreting the functions of these two proteins *in vivo*: TSP may initiate capture of IRBCs in the circulation as the bond is strong but short-lived at fast loading rates which are usually experienced when a circulating cell first comes into contact with the endothelium, as compared to the similar function of selectin in mediating leukocyte migration [27]. On the other hand, CD36 is able to form strong bonds even under slow loading and is insensitive to stress rate change. Such long-lived persistent binding is very likely to form stable attachment once the cells are captured. Similar function has been found in full length cadherin bonds and integrin α_L_β_2_ binding to ICAM-1 [31], [32]. Both laboratory clones and clinical isolates have been shown to stably bind to purified or cell-expressed CD36 under flow conditions [14], [33], [34] while TSP alone cannot form long-lasting adhesion [14].

**Figure 4:**
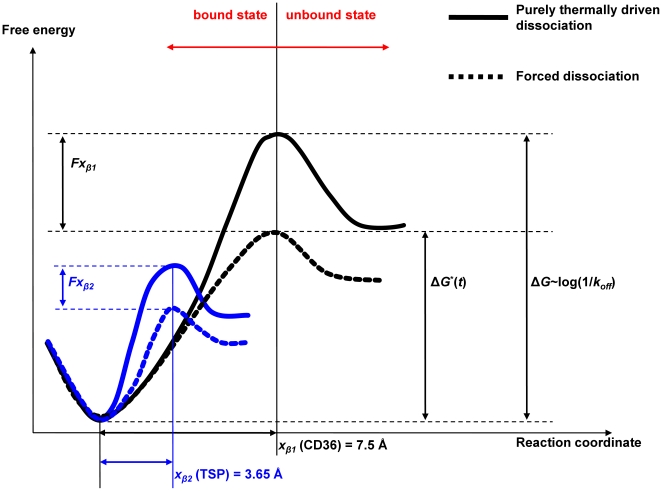
Schematic of energy landscape of CD36 and TSP reconstructed from dynamic force spectroscopy data. Δ*G* is the original energy barrier proportional to log(1/*k_off_*) whereas ΔG* is the force reduced energy barrier. *x_β_* is the reactive compliance or barrier width and *F* the applied force.

By applying Bell-Evans model to the experimentally identified force spectra, the intrinsic chemical potential and energy landscape of the binding complex can be extrapolated. As shown in Fig. 4, the native unstressed state of the bound complex at physiological temperature has an energy barrier with a height of Δ*G*. Such a barrier can be overcome by thermally induced spontaneous dissociation over a natural lifetime of *τ*. In the case of forced unbinding, external mechanical forces decrease the barrier potential by *Fx_β_*. Thus, the more the force is applied, the lower the energy barrier becomes and finally the thermal fluctuation causes the bound complex to break. The characterized energy landscapes for the two proteins showed significant differences at physiological temperature. CD36 needs to overcome a much higher intrinsic energy barrier than TSP when no force is applied and thus forms more stable binding at equilibrium. However, when an external force is applied, CD36 is more susceptible than TSP since the energy barrier decay under the same force for CD36 (*Fx_β_*) is about twice that of TSP owing to different *x_β_*. Thus, it is predicted that under a high level of external force, TSP binding becomes more stable than CD36 and is able to capture fast flowing cells.

The most common interaction identified in most of the infected cells appears between the CIDR region of *Pf*EMP1 and CD36 [35], [36]. It has been demonstrated that the recombinant CIDR peptides are able to block and even reverse the interaction between infected cells and CD36 [37]. Here, we characterized the force spectra of both CD36-IRBCs interaction and CD36-CIDR interaction. At a given loading rate, CD36-CIDR interaction force is consistently smaller than that of CD36-IRBCs interaction. This may be attributed to the recombinant CIDR being conformationally different from that of the native binding domain on the cell surface, and thus, exhibited weaker binding as observed. It is also likely that the CD36-CIDR interaction is a subset of CD36-IRBCs interaction, or in other words, CIDR is involved in CD36-IRBCs interaction but is not the sole ligand (or moiety of *Pf*EMP1) involved. This speculation is also supported by the results from our control experiment, in which excess CIDR reduces but not eliminate the binding between CD36 and IRBCs. It has been shown in the literature that CD36 may have multiple binding domains involved in interacting with IRBCs [38], [39], [40], [41] and several molecules on the surface of IRBCs may interact with CD36 besides *Pf*EMP1, such as phosphatidylserine [42] or modified band 3 [43]. Different molecules or different moieties of *Pf*EMP1 may act synergistically or in concert to determine the overall CD36-IRBCs interaction strength. Further studies will be needed to explore the other molecular target(s) to facilitate the development of cytoadherence inhibitors against CD36 mediated bonding.

Although the presented simplified *in vitro* molecule-cell interaction scheme may not fully reflect the complexity of *in vivo* cell-cell interaction, our fundamental measurements of the kinetic properties of individual cytoadherent molecules no doubt can provide new insights into the molecular basis of cytoadherence and help to characterize the key molecular interactions involved in pathology of malaria disease, which may lead to further development of innovative therapeutic methods.

## Materials and Methods

### Parasite culture

Intraerythrocytic stages of *Plasmodium falciparum* 3D7 were cultured *in vitro* according to the conventional method [44]. Parasites were grown in human erythrocytes in RPMI 1640 medium (Invitrogen Singapore Pte Ltd, Singapore) supplemented with 0.5% Albumax I (Invitrogen), 2 mM L-glutamine, 50 µg/ml hypoxanthine and 25 µg/ml gentamycin at 2.5% hematocrit with a gaseous phase of 5% CO_2_, 3% O_2_, and 92% N_2_ at 37°C. Synchronization of the culture was achieved by sorbitol treatment [45].

To select the mature-stage parasites (late trophozoites and schizonts) which have knobs on the cell surface, the mature-stage parasite culture was allowed to bind to a platelet-coated glass Petri dish [46] for 1 hour at 37°C inside a glass candle jar and the unbound cells were washed away gently using RPMI 1640. The infected erythrocytes which remain bound to the platelet-coated Petri dish have surface knobs which can bind to CD36 on the surface of platelets. The bound cells can be released after the re-invasion and collected for continuous culture. Knobby phenotype of the culture was continuously monitored by means of AFM scanning method [10].

MACS method was applied to enrich the late stage infected cells from cultures [47]. After enrichment, the infected cells would constitute 80∼90% of total cells as identified by optical microscopy method, this would significantly facilitate the subsequent force measurement.

#### Expression and purification of recombinant CIDR peptide

The primers used were 5-CCGGAATTCGAAGACAAAATTATGTCCTATAA-3 as the sense primer and 5- CTAGTCTAGATTAGGAGCGGGCGACACTTCT-3 as the anti-sense primer. EcoR I and Xba I endonuclease sites were included in these two primers respectively to facilitate subsequent cloning steps. PCR products digested with EcoR I and Xba I were inserted into the modified pET32a vector which contains an N-terminal His_6_ or GST tag. Positive clones were screened by using PCR and then confirmed by DNA sequencing.

Sequence confirmed constructs were transferred into BL21 to express the recombinant peptides. For protein expression, LB medium was inoculated with a stationary phase culture of BL21 grown until the OD600 was 0.6. IPTG was added to a final concentration of 0.3 mM, and cultures were grown overnight at 18°C. Protein purification was carried out under native conditions using HisPur Cobalt Resin (Thermo Scientific, IL). SDS-PAGE was performed to analyze the protein expression and purity.

#### AFM Tip Functionalization

Fresh silicon nitride tips (model MLCT, Veeco Instrument, Santa Barbara, CA) were cleaned in ozone plasma for 30 min in a UV cleaner (Veeco Instrument). The cleaned tips were then incubated in 1 mg/ml BSA in PBS for overnight at 4°C. BSA coated tips were first immersed in 2 mg/ml BS^3^ PBS solution (Pierce Biotechnology, Rockford, IL) for 30 min and then in 1 mg/ml NH_2_ terminated bifunctional PEG (Pierce Biotechnology) solution for 2 hours. PEG coated tips were again immersed in 2 mg/ml BS^3^ PBS solution (Pierce Biotechnology) for 30 min and then in 20 µg/ml recombinant CD36 (amino acids Gly30 to Asn439 of human CD36, R&D systems, Minneapolis, MN) or purified TSP (Sigma-Aldrich, St. Louis, MO) PBS solutions for another 2 hour. Finally, the extra free activated amine groups were quenched in 1 mg/ml Glycine PBS solution (Sigma-Aldrich) for 30 min and the functionalized tips were ready to use immediately. To bind GST-fusion CIDR peptide to the AFM tip, BS^3^ activated PEG coated AFM tip was first incubated in 100 µg/ml mouse anti-GST antibodies (Invitrogen) for 2 hours, washed in 1 mg/ml Glycine PBS solution (Sigma-Aldrich) for 30 min and then incubated in 200 µg/ml CIDR peptide for another 1 hour. In antibody blocking experiments, the functionalized tip with CD36 was immersed in 50 µl anti-CD36 (100 µg/ml) (clone SMφ, Abcam Inc., Cambridge, USA) for half an hour and gently washed in PBS. In CIDR blocking experiments, free His_6_ tagged CIDR peptides (to a final concentration of 200 µg/ml) were directly added into the solution where the tip and cells were immersed in for half an hour before further force curve collections were started again.

#### Substrate preparation

13 mm coverslip substrates were coated with lectin molecules which strongly bound to carbohydrate molecules and glycoproteins on the cell surface [48]. The glass substrates were first silanized by treating with gas form of APTES following the methods in the literature [49]. PHA-E lectin molecules (Sigma-Aldrich) or CD36 were crosslinked to APTES treated glass through similar surface chemistry modification as functionalization of the AFM tips.

#### Sample preparation

The magnetically enriched cells (over 80% pigmented late stage IRBCs) were incubated on the PHA-E coated coverslip for 10 min before free and loosely attached cells were gently washed by rinsing in medium for three times.

#### AFM Data Collection

A MultiMode PicoForce system equipped with NanoScope IV controller (Veeco Instruments) was used to measure the adhesion forces between CD36 or TSP functionalized tips and IRBCs. Samples were held in a fluid cell and the local environmental temperature could be adjusted through a temperature controller. Triangular silicon nitride cantilevers (model MLCT-AUNM, Veeco Instrument) with nominal spring constant of 0.01 N/m were used for functionalization and force curve collection. The spring constant of each functionalized cantilever was calibrated *in situ* by the nondestructive thermal tune method before each experiment using the build-in function provided by the software Nanoscope 6.14 (Veeco Instruments). A 50X objective lens was mounted to the optical microscope to help to confirm the infected cells by identifying the hemozoin that were seen as black dots inside the cells. In the force mode, setting parameters (ramp size around 2–5 µm, contact force in relative trigger mode around 50–300 pN and contact time around 50–300 ms) were adjusted accordingly to obtain reproducible single-peak specific interaction force curves at low frequency (<30%). This would provide confidence on the rupture mainly from a single bond [50]. All the approaching velocities were set at constants of 2–5 µm/s and the pulling speeds were adjusted within the range of 0.1 µm/s to 15 µm/s to scan over a range of loading rates for constructing the force spectra. The force-extension curves for each approach-contact-retract cycle were monitored on the screen and captured continuously. At least three different locations on each infected cell were tested for 100–200 cycles at each pulling velocity to collect a set of adhesion curves with a binding frequency varying between 5–40%.

When the temperature control was switched on, the whole system as well as the sample was left for equilibrium for at least 30 minutes before the experiments were conducted.

All experiments at the same conditions were repeated on different cells and using different tips. The control experiments were repeated at least for three times and at least 100 binding force curves were collected for each control experiment. The *P*-values were calculated using student t-test in Minitab (Minitab Inc., PA).

#### Force Curve Analysis

**Figure 5:**
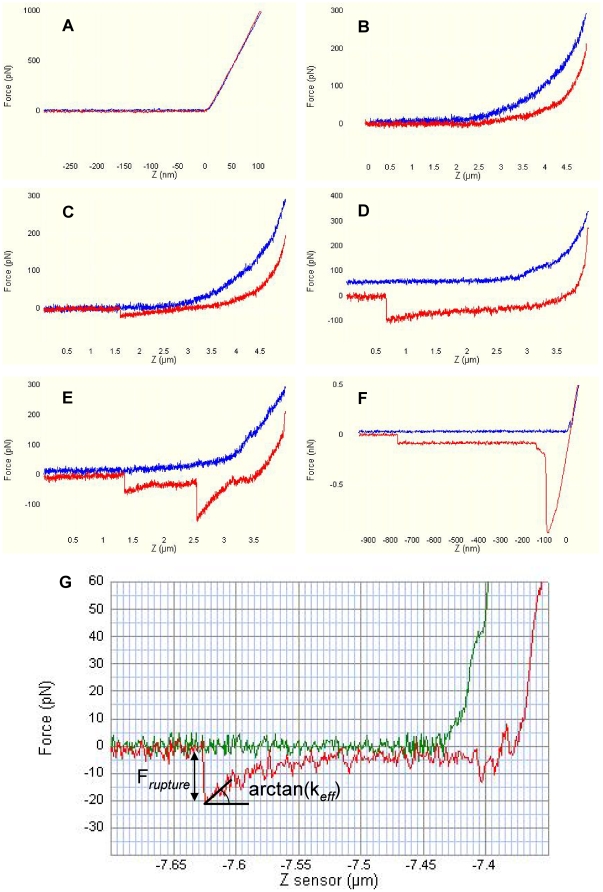
Representative profiles of the force curves obtained. (A) force-displacement curve obtained on a hard substrate surface (B) force-displacement curve obtained on soft cells (C) a specific single peak rupture at low pulling velocity (D) specific single peak rupture at high pulling velocity, gap between the zero lines of approaching and retraction traces was due to hydrodynamic force induced on the bending of the cantilever (E) specific multi-peaks ruptures, only the last peak was assigned as single bond rupture (F) planar extension curves indicating tether formation (G) an example force curve with rupture force and effective bond stiffness identified.

Only the standard shaped force curves with specific unbinding events would be considered for further analysis. Typical examples of force-distance curves collected from the experiments are shown in Fig. 5. When a bare tip was tested on hard mica surface (Fig. 5A), typically there was a sharp transition from horizontal line to a constant slope indicating the contact point in approaching trace and there was no interaction or rupture peak in retraction trace. When a bare or functionalized tip was tested on soft normal cells (Fig. 5B), the transition in approaching trace was smooth and continuous due to deformation of cells but usually no interaction was formed. Usually the indentation part in the approach and retraction traces obtained on soft cells did not overlap because of viscoelastic properties of cells. When a functionalized tip was tested on infected cells (Fig. 5C, D, E and F), approaching trace was similar to that on normal cells but in some curves, the retraction trace showed nonlinear extension below zero line followed by a sharp rupture peak. In such curves, the magnitude of the peak was assigned as the rupture force (*F_rupture_*) and the gradient in the extension immediately before rupture was assigned as k*_eff_*, which was the effective bond stiffness used to calculate the loading rate (k*_eff_* multiplied by the pulling speed) experienced by the bound complex in the particular unbinding event (Fig. 5G). The rupture forces obtained at different retraction velocities showed different magnitude (Fig. 5C and D) and there were gaps between the zero lines in the approach and retraction traces in the curves captured at fast speed compared to the overlapped zero lines captured at slow speed. This was because of the hydrodynamic drag acting on the cantilever in the fast pulling. Also there could be either one or multiple peaks in one curve (Fig. 5E), which was determined by the functional groups available on the tip and the density of bonds formed in the contact area. In the fraction of force curves with multiple peaks, only the last peak was used to construct subsequent histograms for single bond strength [51]. Also, since the total binding frequency (defined as the number of force curves showing binding events out of the whole set of force curves) was controlled around and below 30%, we were confident that most of the force curves resulted from single bond rupture [49], [50] and would not be affected by the coating density of the receptors on the tip. Interestingly, there was a subset of force curves collected which showed planar extension before rupture. This is possibly due to tether formed from red cell membrane (Fig. 5F). In such a case, external force acting on the molecular bonds remained constant and the ruptures of the bonds mainly resulted from thermal fluctuation under zero loading rate. Therefore these curves were excluded from analyses in the current study.

#### Statistical analysis

For each force curve showing specific rupture, bond strength (*F_rupture_*) and loading rate (the product of *k_eff_* by the retract velocity) (Figure 5G) were determined using a home-build code written in MATLAB (The MathWorks, MA, USA). To construct the force spectra under certain conditions, about 1,000–2,000 force curves with specific rupture events were collected over 10–15 different velocities which covered 3–4 orders of loading rates (roughly ranging from 10 to 10,000 pN/s). Biophysical kinetic parameters were characterized by applying Bell-Evans model to fit the experimental data [23] analyzed using the binning method developed by Hanley et al., [52] Panorchan et al. [53] and Dobrowsky et al. [20]. The overall force spectra was partitioned by using binning windows of 100 pN/s for loading rates between 100 and 1,000 pN/s and by binning windows of 1,000 pN/s for loading rates between 1,000 and 10,000 pN/s and so on. Each bin yielded a peak force by Gaussian fitting. By plotting the peak force as a function of mean loading rate for each bin, the unstressed dissociation rate and the free reactive compliance for the molecular interactions were extracted.

*P* values indicating statistical significance were calculated using student t-test for comparison of two populations or one-way ANOVA and Tukey test for comparison of more than two populations simultaneously. *P*<0.05 was considered as statistically significant.
