# Correction: Molecular Mechanistic Insights into the Endothelial Receptor Mediated Cytoadherence of *Plasmodium falciparum*-Infected Erythrocytes

**DOI:** 10.1371/annotation/4aae1ede-d539-40d5-bcc2-5790b1709fde

**Published:** 2011-03-24

**Authors:** Ang Li, Tong Seng Lim, Hui Shi, Jing Yin, Swee Jin Tan, Zhengjun Li, Boon Chuan Low, Kevin Shyong Wei Tan, Chwee Teck Lim

The authors would like to add the following to the Acknowledgments section: "We would like to thank Dr Brian M Cooke (Monash University) for providing GST fused CIDR peptide and useful discussion. We would also like to acknowledge support from the Singapore-MIT Alliance for Research and Technology (SMART), Singapore-MIT Alliance (SMA), Global Enterprise for Micro Mechanics & Molecular Medicine (GEM4) and Mechanobiology Institute, NUS."

